# Mucin Thin Layers: A Model for Mucus-Covered Tissues

**DOI:** 10.3390/ijms20153712

**Published:** 2019-07-29

**Authors:** Valeria Rondelli, Emanuela Di Cola, Alexandros Koutsioubas, Jenny Alongi, Paolo Ferruti, Elisabetta Ranucci, Paola Brocca

**Affiliations:** 1 Department of Medical Biotechnologies and Translational Medicine, Università degli Studi di Milano, L.I.T.A., Via F.lli Cervi 93, 20090 Segrate, Italy; 2 Jülich Centre for Neutron Science (JCNS) at Heinz Maier-Leibnitz Zentrum (MLZ), Forschungszentrum Jülich GmbH, Lichtenbergstrasse 1, 85748 Garching, Germany; 3 Department of Chemistry, Università degli Studi di Milano, Via Camillo Golgi 19, 20133 Milano, Italy

**Keywords:** mucin, mucus, model membrane, X-ray scattering, neutron reflectivity, quartz crystal microbalance, amphoteric polymers, polyamidoamine

## Abstract

The fate of macromolecules of biological or pharmacological interest that enter the mucus barrier is a current field of investigation. Studies of the interaction between the main constituent of mucus, mucins, and molecules involved in topical transmucoidal drug or gene delivery is a prerequisite for nanomedicine design. We studied the interaction of mucin with the bio-inspired arginine-derived amphoteric polymer d,l-ARGO7 by applying complementary techniques. Small angle X-ray scattering in bulk unveiled the formation of hundreds of nanometer-sized clusters, phase separated from the mucin mesh. Quartz microbalance with dissipation and neutron reflectometry measurements on thin mucin layers deposited on silica supports highlighted the occurrence of polymer interaction with mucin on the molecular scale. Rinsing procedures on both experimental set ups showed that interaction induces alteration of the deposited hydrogel. We succeeded in building up a new significant model for epithelial tissues covered by mucus, obtaining the deposition of a mucin layer 20 Å thick on the top of a glycolipid enriched phospholipid single membrane, suitable to be investigated by neutron reflectometry. The model is applicable to unveil the cross structural details of mucus-covered epithelia in interaction with macromolecules within the Å discreteness.

## 1. Introduction

Mucus is a highly viscoelastic secretion, covering the epithelia surfaces of the gastrointestinal, pulmonary, oral, nasal, and genital tracts. Its function and composition differ at different locations of our body, but the general task of mucus is to protect mucosal tissues from dehydration, mechanical stress, and to act as barrier against microorganisms and toxic substances [1]. Mucus is mainly composed of water (up to 95%), lipids, small proteins, and nucleic acids, but its mechanical and viscoelastic properties are due to the presence of high molecular weight (MW) glycoproteins, identified as mucins (MW from 0.5 KDa to 200 MDa). Mucins are highly glycosylated proteins where sugars can account for up to 80% of the MW. Sugar chains of few to tenth units form brushes extending out from the amino-acidic backbone, along which they are regularly distributed. The sugar composition consists of galactose, fucose, *N*-acetylgalactosamine, *N*-acetylglucosamine, and sialic acid, the last bearing negative charges. Specifically, the flexible glycosylated core of the protein (mainly composed of serine and threonine residues) ends with hydrophobic domains enriched in cysteine residues. The latter can form inter-molecular bonds via disulphide links, promoting mucin association. 

Mucin can establish adhesive interactions with particulates via electrostatic interactions, van der Waals forces, hydrophobic forces, hydrogen bonding, or chain entanglement. Little is still known about the interactions between mucin and other macromolecules [2,3]: they can either penetrate rapidly or establish prolonged contact with mucus, depending on their specific composition and structure. 

Studying the behavior of nanomedicines in mucus models is an essential preliminary investigation in topical drug delivery. Interactions are usually investigated in vitro by colloidal stability and turbidimetry methods, and more deeply, the interaction of nanoparticles with mucus in bulk can be studied by X-ray and/or neutron small angle scattering techniques, sensing structures at the nanoscale [4]. However, beside studying complex multicomponent nanovectors to be applied in delivery, the interaction of nanovector single components with mucus is also a relevant matter of investigation. On one side, the fate of the delivery agents is often determined by the interaction of the interface components with the biological barrier. Moreover, vector components after administration remain in the mucus gel where the clearance processes occur [5], the effectiveness of which (may) depend [6,7] on their attitude to interact with the mucus bio-gel. In fact, a fundamental process in lung mucus biology is the mucociliary clearance, a lung’s primary defense mechanisms which impairment is involved in human diseases such as chronic obstructive lung disease, idiopathic pulmonary fibrosis, cystic fibrosis, and primary ciliary dyskinesia. In this process, the mucus layer, typically about 10 microns thick, is continuously removed. Particles deposited in the airways are cleared out by mucociliary clearance, usually within 24–48 h [8]. In this perspective, macromolecules intended to interact or to penetrate the mucus layer deserve to be investigated in their attitude to get caught in the mucus mesh or in their possible role of mobility promoter or mobility antagonist inside the mucus layer. Notably, the interest in studying polymer interaction with mucus overcomes the specific field of drug delivery system design [9,10,11,12]. On this aspect, profitable hints at the molecular scale can come from the study of thin layer model systems mimicking the biological conditions [13]. For this purpose, model systems very often involve the use of commercial pig gastric mucin (PGM) as a mimetic system of mucus, having mucins play a major role in determining stickiness and viscoelasticity of the mucus barrier. 

We worked on the development of thin layer mucus model environments to deepen the understanding of mucin interactions with biomolecules. 

We focused our study on the d,l-ARGO7 polymer, a promising linear polyamidoamine (PAA) that may be of interest for drug and genetic material delivery. PAAs constitute a polymer family obtained by polyaddition of prim-monoamines or bis-sec-diamines with bisacrylamides [14,15]. PAAs are intrinsically cationic, containing a tert-amine group in the main chain, however, at physiological pH, most of them bear an overall ionic charge significantly lower [16] than other widely used polycations, such as poly-l-Lysine (PLL) and polyethyleneimine (PEI). Therefore, they are usually less toxic than PLL and PEI [17]. Moreover, unlike chitosan, PAAs are usually water soluble and hydrolytically degradable in aqueous media at pH ≥ 7.0 even without intervention of external factors such as enzymes or strong alkalies. Carboxyl substituted amines or bisacrylamides give amphoteric PAAs, which are normally highly cytobiocompatible [16,17]. Natural α-amino acids, including arginine, behave similarly [18,19,20,21], with the additional property of being chiral. Notwithstanding, the chiral centers are not a part of the main chain but are part of the side substituents. Therefore, PAAs assume pH-dependent stable conformations in aqueous systems. In particular, the polyaddition product of d-, l-, and d,l-arginine with *N*,*N*′-methylenebisacrylamide, nicknamed d-, l-, and d,l-ARGO7, have been reported [18,19]. These polymers are amphoteric with isoelectric point 9.7 and features, on average, +0.25 charge per repeat unit at pH 7.4, with no detectable influence by their chirality. In vitro tests with mouse embryo fibroblasts balb/3T3 clone A31 showed that L-ARGO7 is endowed with cell internalization ability and little cytotoxicity. It was speculated that all arginine-rich synthetic ARGO7 stereoisomers might share the cell-permeating properties of polyarginines, but without the toxic character of the latter. ARGO7 isomers were considered potential non-toxic mucin interacting polymers [18]. 

The primary characterization of the interaction in the bulk was obtained by small angle X-ray scattering (SAXS); then, thin layered mucin depositions were investigated by quartz crystal microbalance with dissipation (QCM-D) and neutron reflectometry (NR), evaluating their potentiality as further platform for interaction studies. Of note, as a final step, we developed a quite complex mimic for the mucus-covered epithelial surface, consisting of an asymmetric model membrane containing glycolipids, on the top of which lies a thin mucin layer. In fact, complexation between mucins and biomacromolecules may take place close to the cell membrane surface, as reported, for instance, in a class of in vitro experiments on chitosan–mucin complexation [22], and the model proposed is suitable for the investigation of nano-sized carriers delivery and membrane interaction. This last model system is investigated by NR. 

The properties of d,l-ARGO7 made it a good candidate to prove the applicability of our model systems and of the investigation techniques applied, allowing to unveil that the polymer has a peculiar role in affecting mucus structure. 

## 2. Results and Discussion

### 2.1. Mucin in Bulk: Small Angle X-ray Scattering (SAXS)

**Figure 1:**
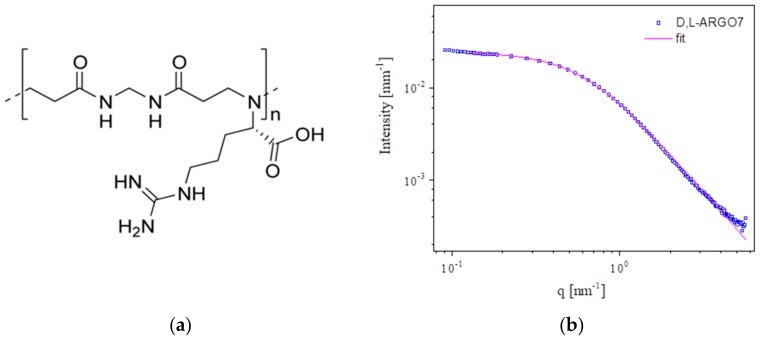
(**a**) Chemical structure of d,l-ARGO7. (**b**) SAXS intensity profile of d,l-ARGO7 at pH = 6.8 and 135 mM NaCl. Blue squares refer to the experimental data, while in pink it is reported the best fit by a Gaussian coil polymer chain model of radius of gyration 2.5 nm.

The synthetic d,l-ARGO7 polymer (Figure 1a) is positively charged at physiological pH. Hence, it is suitable to determine electrostatic interaction with the mucin carbohydrate chains that bear 0.5–1.5% bounded negatively charged sialic acid in the case of Type III pig gastric mucin (PGM). d,l-ARGO7 10 mg/mL water solution shows a SAXS spectrum well reproducible by a Gaussian polymer model described in the Material and Methods in Equation (6), giving a radius of gyration of 2.5 nm (see Figure 1b). SAXS spectra of mucin was in agreement with data in the literature [23,24,25].

**Figure 2:**
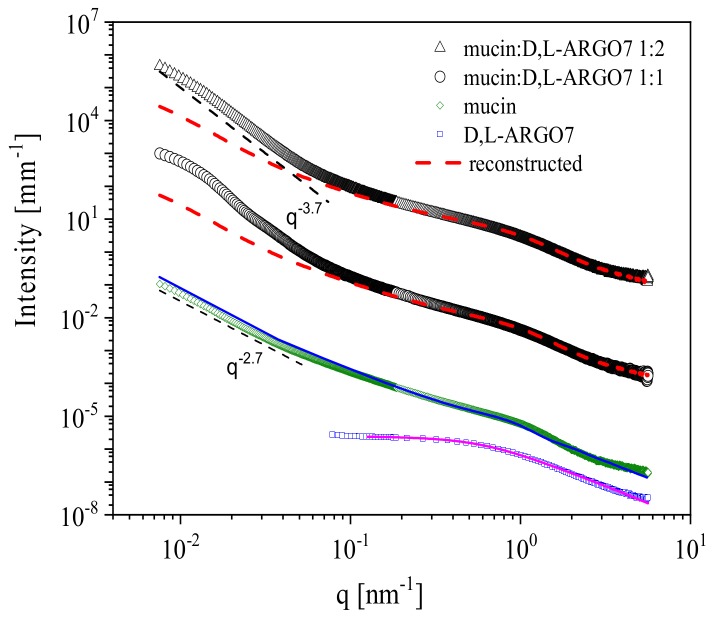
SAXS intensity profiles of the individual d,l-ARGO7 polymer and PGM mucin and of PGM/ d,l-ARGO7 mixed solutions at pH = 6.8 and 135 mM NaCl. From top: 1:2 weight ratio, black triangles not rescaled [in absolute units], and 1:1 polymer/mucin ratio, black circles, rescaled ×10^−3^, bare PGM (10 mg/mL) in buffer with fit (blue line) according to dumbbell-like shape form factor [23,24,25], green diamonds, rescaled ×2 × 10^−3^; and d,l-ARGO7 (10 mg/mL), blue squares rescaled ×2 × 10^−4^, with fit (pink line) by a Gaussian coil polymer chain model of radius of gyration 2.5 nm. Red lines represent the profiles reconstructed by summing the two components according to weight ratio.

We investigated the structural outcome of d,l-ARGO7-mucin interaction in solution. We considered mixed systems of d,l-ARGO7 in mucin 10 mg/mL solutions at physiological condition (PBS 135 mM NaCl). SAXS was acquired for mixed PGM/ d,l-ARGO7 solutions at two different weight ratios: final mucin concentration was 1% in the two samples, while d,l-ARGO7 was added to the solutions in weight ratio 1:1 and 1:2, respectively, by mixing a previously dissolved stock solution. Figure 2 reports the scattering profiles of the two mixed systems in weight ratio 1:2 (empty triangles) and in weight ratio 1:1 (empty circles), together with the SAXS intensity profiles of mucin (empty diamonds) and of the free d,l-ARGO7 polymer (empty squares). In Figure 2 we compare the SAXS intensity of the mixed states with the sum of the experimental scattering intensity of the single components (red dotted lines) as:

(1) $$I_{mixed}\left(q\right)=I_{PGM}\left(q\right)+A\times I_{ARGO}\left(q\right)$$

where A = 1 for PGM/d,l-ARGO7 weight ratio 1:1 and A = 2 for PGM/d,l-ARGO7 weight ratio 1:2. At low-q, a significant deviation between the model and the experimental SAXS data occurs. On the contrary, in the higher-q range, corresponding to 0.1–10 nm associated distances, the scattered intensity is not far from the sum of the intensities of the two individual components. Overall, these features are consistent with the presence of some large-scale objects formed in the mucin mesh that originates from aggregates of bounded polymer and mucin, not involving a large quantity of the material. In fact, the slope of the profile approaching the lowest q range shows a power low decrease with an exponent of −3.7, typical of a surface fractal dimensionality, consistent with the presence of phase separated large clusters in the mesh. The deviation of this value from the lower one, −2.7 (see Figure 2), typical of mass fractal gel networks [26], indicates a quite tight interaction among the components within the clusters. An estimation of the cluster sizes is obtained by a low-q data Guinier analysis giving a Rg ≥ 185 nm for R1 and Rg ≥ 209 nm for R2 [27]. Although at 135 mM salt concentration a strong screening of charges is expected, electrostatics may play a major role in stabilizing oppositely charged d,l-ARGO7 and mucin interaction. Moreover, as shown for other cationic biopolymers (like chitosan), other types of interactions might also occur, such as, for instance, hydrogen bonding, which can favour entanglement formation among the two polymers [28,29]. However, the SAXS intensity profile of the mixed state cannot be simply reconstructed by the sum of the intensities relative to the clusters, free mucins, and polymers scattering contributions [30]. This suggests that some more loose entanglement occurs between mucin and polymer affecting the mesh structure at the intermediate q range. Different mechanisms have already been proposed to regulate the binding of molecules to mucin [31]. In general, the occurring mechanisms affect the fate of the particles facing and entering the mucus barrier and their mutual interaction may allow tuning the mucus gel properties, and strengthening or weakening the gel mesh [32,33]. 

### 2.2. Mucin Thin Films

#### 2.2.1. Quartz Crystal Microbalance with Dissipation (QCM-D)

In a QCM-D experiment, the sensor is sandwiched between a pair of electrodes and it can be excited to oscillate in the thickness–shear mode at its fundamental resonance frequency (*f* ≈ 5 MHz) and odd overtones (*n* = 3, 5, 7, 9, 11) by the application of an alternating voltage. The resonance frequency depends on the total oscillating mass of the sensor and sensor surface adhering layers, including coupled water. The Q-Sense software determines the resonance frequency and the decay time, τ_0_, of the exponentially damped sinusoidal voltage signal over the crystal, caused by switching off the voltage applied to the piezoelectric oscillator. This allows it to acquire the dissipation factor, *D*, via the relation:

(2) $$D=\frac{1}{f_{\tau_{0}}}$$

Classically, the Sauerbrey relationship is used for quantitative determination of mass deposited on the sensor surface, where the mass sensitivity constant, *C*, is equal to 17.7 ng/(cm^2^Hz) at *f* = 5 MHz. 

(3) $$\Delta m=-C\frac{\Delta f}{n}$$

However, such a relationship has been derived for uniform thin rigid films with material properties indistinguishable from those of the crystal resonator. For soft thick films, the Sauerbrey equation is no longer valid and needs to be corrected for the influence of the medium and the viscoelasticity. 

**Figure 3:**
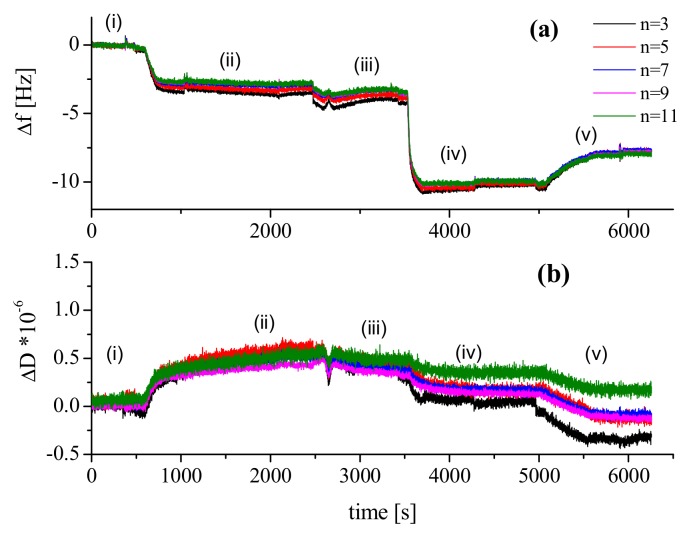
Time dependence of (**a**) the frequency (Δ*f*) and (**b**) dissipation (Δ*D*) shifts. The raw data are normalised for the overtone number. Numbers of overtones are presented in the legend.

Here, the experimental procedure for the QCM-D measurements was the following: (i) PBS solution (pH 7.4) was injected at time t_0_ to get a baseline; (ii) injection of PGM with known bulk concentration (*c* = 20 mg/L) at time t_1_ with the adsorption kinetics typically followed on-line during 15 min; (iii) injection of PBS solution at time t_2_ and 3-times exchange of the chamber volume to remove the protein substance that was not surface confined; (iv) injection of polymer solution (d,l-ARGO7 c = 100 mg/L) at time t_3_; (v) additional rinsing step in PBS at time t_4_ with 3-times exchange volume as before. The flow rate (0.1 mL/min) was stopped after each injection. Measurements were performed at T = 25 °C. In Figure 3, the raw data (Δ*f* and Δ*D* versus time) are shown. 

The first absorption process (labelled (ii)) corresponds to the absorption of PGM onto the surface. As previously reported for bovine submaxillary gland mucin (BSM), the adsorption of PGM forms an initially rigid layer as concluded from the small increase in Δ*D* (Δ*D* ≤ 0.5 × 10^−6^) [34]. A decrease in Δ*f* is observed, corresponding to low mass absorption of about 55 ng/ cm^2^, estimated according to equation (2). Such a value is in agreement with that reported by Cardenas et al. [35] for MUC5B films. The deposited layer, even if thin, was found to be very stable, and no desorption has been seen upon rinsing with buffer in process (iii).

Although the Sauerbrey is an approximation, we can provide an estimation of the thickness *d* of the PGM layer on the SiO_2_ support from the value of the absorbed mass ∆m, assuming for the mucin layer density ρ a value of 1.05 g/cm^3^ (hydrated sugars), as reported in the literature [36]:

*d* = ∆*m*/*ρ* (4)

This brings to a value for the layer thickness of about 20 Å.

In process (iv), d,l-ARGO7 (*c* = 100 mg/L) was injected in the cell. The kinetics of absorption was rapid, and equilibrium was reached in less than 5 min. The absorption process results in a Δ*f* of −6 (Figure 3a) but no significant changes ((ΔD changes ≤ 0.5 × 10^−6^) are simultaneously registered for the dissipation (Figure 3b) during this process. Nevertheless, the harmonics seem to no longer overlap and a small decrease of the dissipation is observed, indicating that the presence of the polymer might perturbate the PGM layer. This trend is more evident after rinsing with PBS buffer in process (v), where desorption is registered (mass lost in Figure 3a). At the same time, the greatest change in dissipation is observed for the third harmonic (*n* = 3, black line, Figure 3b) which probes far from the chip surface, suggesting that polymer has bound to the PGM surface, changing the layer structure. During the rinsing process, we observed some material loss which may be attributed to the flushing of the complexes of PGM/d,l-ARGO7 that have been observed by SAXS investigation [37].

#### 2.2.2. Neutron Reflectivity (NR)

To deepen the structure of the PGM deposited layer and the detailed effects brought by the polymer to it, we applied neutron reflectivity, which allows investigation of the transverse structure of layered samples within few Å sensitivity.

**Figure 4:**
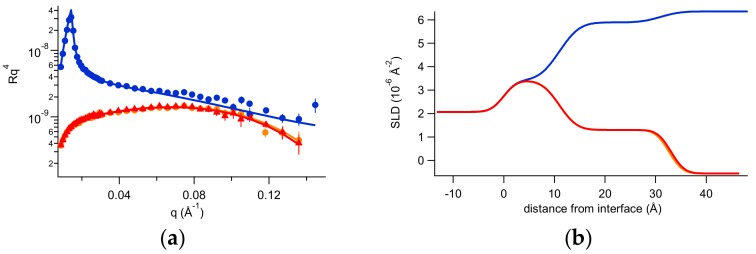
(**a**) Reflectivity curves (symbols), relative best fits (lines), and (**b**) obtained scattering length density (SLD) profiles of the mucin layer investigated in H2O NaCl (orange), in D2O (blue), and after the last flushing in H2O (red) at room temperature.

Subsequent to the characterization by neutron reflectivity of silicon/solution interface, 0.2 mg of PGM were injected in the measuring cell (6 mL total volume) in 150 mM NaCl H_2_O solution at room temperature. After a waiting time of 30 min for system equilibration, reflectivity was measured, then 150 mM NaCl H_2_O was gently flushed in the cell and reflectivity was measured again. Next, 150 mM NaCl D_2_O was gently flushed in the cell and reflectivity was measured, for a full characterization of the system, which was finally brought again in 150 mM NaCl H_2_O to investigate its stability. In Figure 4 NR spectra are shown, demonstrating that the system is very stable, in accordance with previous QCM-D characterization. Fitting parameters can be found in Table S1 of Supplementary Materials.

The described experiments were also useful to define the features of the thin mucin layer deposited on the rigid support. In fact, mucin contains a large amount of sugars, which moieties bring labile hydrogens belonging to -OH and -NH groups. Hence, these labile hydrogens may exchange with H and D atoms of the solvent (H_2_O or D_2_O). Then, the % amount of H and D of the deposited mucin layer depends on the H and D % amount in the hydration solvent, that is, the contrast of mucin depends on the degree of deuteration of the solvent. By fitting, the neutron scattering length density (SLD) for the deposited mucin layer has been found to be about 2.5 × 10^−6^ Å^−2^ in H_2_O and 5.6 × 10^−6^ Å^−2^ in D_2_O.

NR data are in agreement with QCM-D results: the two contrasts’ best fit in fact indicates that a 22 Å thick layer is formed, where mucin had a volume occupation of roughly 60%.

**Figure 5:**
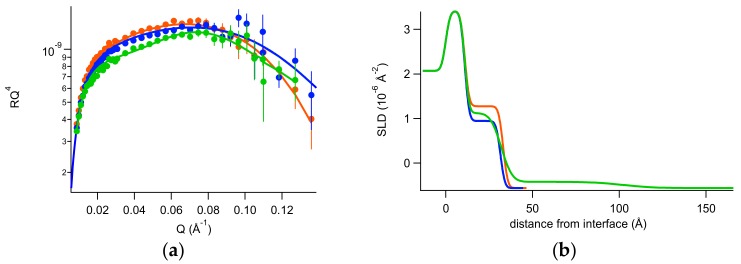
(**a**) Reflectivity curves (symbols), relative best fits (lines), and (**b**) obtained SLD profiles of the mucin layer investigated in H2O NaCl before (orange) and after (green) the incubation of d,l-ARGO7. Blue data correspond to the final system, investigated after flushing.

After the thin mucin layer characterization, we investigated the behavior of the d,l-ARGO7 polymer in interaction with the deposited mucin. We injected in the measuring cell 100 μL of a 2 × 10^−7^ mg/mL of polymer solution in 150 mM NaCl and after 30 min for system equilibration, reflectivity had been measured. Indeed, structural changes to the PGM layer had been observed after polymer injection, as shown by the variation of the reflected profiles in Figure 5a, changing from the orange to the green spectra. Figure 5 additionally shows the profile obtained after a final gentle flushing of 150 mM NaCl water buffer in the measuring cell, performed to elucidating the extent of polymer interaction with the mucin layer.

Best fits to NR data and relative SLD profiles are also reported in Figure 5a,b, respectively, while structural parameters are reported in Tables S2 and S3 of Supplementary Materials.

Results show that, after injection in the measuring cell and equilibration, the polymer forms a highly hydrated 70 Å thick layer on top of the mucin layer and weakly destabilizes it (5% increase of volume of solvent). Furthermore, by rinsing with buffer solution, the polymer was flushed away, and the mucin layer was found to be altered: its thickness was lowered to 20 Å and the included solvent increased up to a value of 50% volume occupation.

These findings agree with joint SAXS and QCM-D results, enforcing the hypothesis that the polymer forms stable complexes with mucin and that the formed complexes phase separate from the mucin mesh. Thus, the rinsing procedure can cause the removal of a quantity of deposited material that is no longer entangled with the remaining layer. 

### 2.3. Mucus-Covered Epithelium Model by Neutron Reflection

Further, we developed a model for mucus-covered epithelial cells, which potentially is of great importance for mimicking physiological environments for trans-mucosal delivery to tissues. In fact, vehicles for the delivery must cross the mucus barrier first and face the problem of membrane inking and crossing only after interaction with mucus, which represents a further complication for their design.

The interaction of mucin with phospholipid membrane has been poorly investigated [38] to date, but recent studies demonstrate that mixing lipid vesicles with mucin results in a lipid membrane surface coated by mucin, where the lipid–mucin interactions are not predominantly of electrostatic nature [39].

On the other hand, neutron reflection has been profitably applied to the investigation of the morphology, structure and stability of similar systems, such as human saliva [40] and lubricin [41].

Here, we report the optimal conditions to deposit stable mucin layers on top of model membranes of increasing complexity in order to mimic different cell membrane portions. We proceeded in two steps: first, PGM was deposited on top of a monocomponent phospholipid membrane prepared by vesicle fusion on the silica support. The model was further modified regarding its complexity by adding anionic glycolipids to the phospholipid membrane, another key component involved in cell signaling. 

For the first model, we deposited by vesicle fusion a d_62_DPPC membrane and, after its characterization in 150 mM NaCl solutions in D_2_O and H_2_O, 0.2 mg of PGM was injected in the measuring cell. Reflectivity has been measured after letting the system equilibrate for 30 min, again in the two contrasts conditions with 150 mM NaCl.

Data with their relative best fits, fit parameters, and relative SLD profiles are reported in Figure S1 and Tables S4 and S5 of SM. The results revealed that the mucin-to-membrane interaction was very poor, and the little change observed in reflectivity spectrum was compatible with weak interaction, limited to only the external lipid headgroups without building up a mucin layer on top of the membrane. 

Therefore, we repeated the experiment incubating PGM on a more complex bilayer system, which is an asymmetric GM1 ganglioside containing model membrane. In fact, we previously showed [42] that the fusion of GM1 containing phospholipid vesicles on silicon substrates generate single membranes with a fully asymmetric disposition of GM1 found only in the outer leaflet membrane, the one exposed to the bulk solvent. Gangliosides protruding out of the bilayer with large sugar head-groups are good candidates for face-to-face interaction with the mucin sugar brushes and, bearing a negative charge, are also susceptible to interact with the cationic polymer.

**Figure 6:**
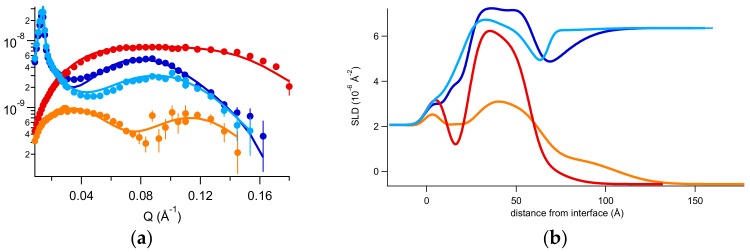
(**a**) Reflectivity curves (symbols), relative best fits (lines), and (**b**) obtained SLD profiles of the d_62_DPPC-GM1 membrane investigated in two contrasts before and after the deposition of the mucin layer on top of it. Color codes: red, membrane in H_2_O NaCl; orange, membrane + mucin in H_2_O NaCl; blue, membrane in D_2_O NaCl; sky blue, membrane + mucin in D_2_O NaCl.

Interestingly, contrary to what was observed for the bare phospholipid membrane, a PGM layer was found on the top of the ganglioside-containing membrane and it was found to be stable under solvent exchange. The spectra with the relative best fits are shown in Figure 6a with the respective contrast profiles (Figure 6b). After interaction with PGM, changes in membrane contrast were observed (lower SLD). For simplicity, in order to fit the experimental data, we accounted for an increase in solvent penetration (30–35% in volume). The latter can be interpreted both as a membrane degradation with consequential removal of part of it from the silica deposition or as an increased lipid specific volume (fluidification) due to the presence of PGM. Both the phenomena are likely to occur. However, we obtained stable membranes with a 30 Å thick stable PGM layer cover. The fit parameters are reported in Tables S6 and S7 of SM.

**Figure 7:**
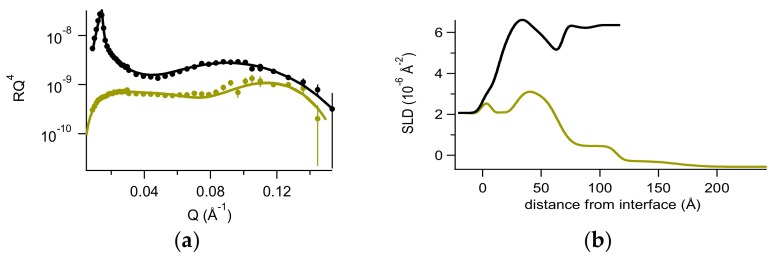
(**a**) Reflectivity curves (symbols), relative best fits (lines), and (**b**) obtained SLD profiles of the d_62_DPPC-GM1 + mucin model system after interaction with d,l-ARGO7 investigated in H_2_O NaCl (olive) and D_2_O NaCl (black).

Further, we investigated the interaction of d,l-ARGO7 with this model system. Data are reported in Figure 7 together with their analysis by fittings NR spectra in two contrasts, the fit parameters of which are reported in Tables S8 and S9 of Supplementary Materials. The results indicate similar effects to those observed in the case of the PGM layer alone: the polymer forms a hydrated thick layer on top of the membrane/mucin system, and part of the material is removed during gentle flushing. Similarly, we observed that flushing results in a weak PGM layer destabilization reflected in membrane thinning and in a loss of compactness. This is probably due to polymer complexation with mucin chains, eventually increasing their mobility upon rinsing. It is worth noting that this outcome is reminiscent of the mechanism of mucus as a defense barrier, couching external agents and allowing their removal by mucociliary clearance.

No effect on the underlying membrane was observed. These results are in agreement with confocal microscopy measurements (data not shown), unveiling that the polymer is internalized in cells by endocytosis.

Interestingly, the underlying membrane was not destabilized by the complex experimental procedure. This finding is very important for the validation of the model here proposed, which aims to mimic in vivo conditions, combining the mucosal environment with the underlying tissue. 

The experiment here reported opens up not only the use of such a model for the investigation of the effects of a variety of active biomolecules, but also to the development of a model platform with increase complexity for even more realistic tissue mimics. This may be reached, for example, by increasing membrane complexity in terms of composition by the addition of sterols and proteins [43,44].

## 3. Materials and Methods 

Type III porcine gastric mucin (PGM) was purchased by Sigma Adrich and used without any further purification. 

1,2-dipalmitoyl-d62-sn-glycero-3-phosphocholine (d_62_DPPC) was purchased by Avanti Polar Lipids. 

Neu5Acα2-3(Galβ1-3GalNAcβ1-4)Galβ1-4Glcβ1Cer (GM1 ganglioside) was extracted and purified according to [45]. 

### 3.1. d,l-ARGO7

Synthesis of d,l-ARGO7. d,l-ARGO7 was prepared as previously reported [18,19]. Briefly: *N*,*N*′-methylenebisacrylamide (1.000 g, 6.23 mmol) and d,l-arginine (1.10 g, 6.3 mmol) were added to water (2.5 mL) under vigorous stirring. The reaction mixture was heated to 55 °C until homogeneous and maintained with gentle stirring for 15 days at room temperature. The terminal double bonds present in the resultant product, if any, were saturated by adding morpholine (25 mg) and kept at room temperature for a further 2 h. The reaction mixture was finally acidified to pH.4, ultrafiltered through a membrane with nominal molecular weight cut-off 3000, and freeze-dried. Yield: 40%. ${\bar{M}}_{w}$ 6800, PDI 1.48.

^1^H NMR (400.132 MHz, D_2_O): d = 1.44–1.72 (br, 4H, CHC**H**_2_C**H**_2_); 2.69 (m, 4H, COC**H**_2_); 3.10–3.20 (m, 4H, C**H**_2_NHC(NH_2_)_2_); 3.30–3.40 (m, 4H, COCH_2_C**H**_2_); 3.60–3.75 (br, C**H**COO^−^); 4.54 (s, NHC**H**_2_NH). 

### 3.2. Membrane Deposition by Vesicles Fusion

Membranes for neutron reflectivity experiments have been deposited by the fusion of lipid vesicles on lipid supports. Both the lipid vesicles preparation protocols and the details of the fusion technique are reported in [42].

### 3.3. Small Angle X-rays Scattering (SAXS)

SAXS investigations were performed at high brilliance ID02 beamline of the European Synchrotron (ESRF, Grenoble, France) [46]. The 2D SAXS patterns were collected using a Rayonix MX-170HS ccd detector. A combination of two samples to detector distances (10 m and 1 m) were employed in order to cover a wide q-range, 7 × 10^−3^ < *q* < 6 nm^−1^. q is the scattering wave vector defined as *q* = (4π/λ) sin ϑ/2, λ being the wavelength (λ ∼ 1 Å) and ϑ the scattering angle. Measurements were performed using polycarbonate capillaries of 2 mm thickness (ENKI, Concesio, Italy) as sample containers. The measured two-dimensional SAXS patterns were corrected for detector artefacts, normalized to absolute intensity scale and azimuthally averaged to obtain the intensity profile *I*(*q*) as a function of q using standard procedures [47]. Spectra were recorded at several positions of the capillary’s length. This allows the ability to control radiation damage that might be induced by X-ray exposure. For each sample, 3–4 frames were acquired, which were subsequently averaged after excluding any possible radiation damage. The background scattering of the PBS buffer (pH 6.8) was also measured by the same procedure and subsequently subtracted from each averaged sample intensity profile using the SAXS utilities analysis package [48].

The SAXS background-subtracted scattered intensity *I*(*q*) can then be expressed as:

*I*(*q*) = *NV^2^Δρ^2^P*(*q*)*S*(*q*) (5)

where *N* is the number of particles per unit volume *V*, Δ*ρ* is the different in the SLD between the particles and the medium, *P*(*q*) and *S*(*q*) are the form and the structure factor, describing the shape and interaction between particles, respectively. In this specific case, we can safely neglect interparticle interactions, i.e., *S*(*q*) ≈ 1. 

The SAXS intensity profile of the free polymer d,l-ARGO7 was modelled using the form factor of a Gaussian polymer coil, given by the following relationship [49]:

(6) $$P\left(q\right)=2\;\left[\exp(-q^{2}R_{g}^{2}\right)+\left(q^{2}R_{g}^{2}\right)-1]/{\left(q^{2}R_{g}^{2}\right)}^{2}$$

where *R*g is the radius of gyration of the polymer chains. 

For q × *R*g < 1, the asymptotic Guinier approximation was used to calculate the radius of gyration:

(7) $$I\left(q\right)=I_{o}\exp\left(\frac{-R_{g}^{2}}{3}\right)$$

The SAXS intensity profiles for the PGM molecules was modelled using a dumbbell-shape form-factor [23,24,25].

*I*(*q*) = *I*(*q*)_peptide_ + *I*(*q*)_globule_ (8)

where

(9) Iqpeptide=I1ξ2q2+I2Ξnqn

and

(10) $$I{\left(q\right)}_{globule}=NV^{2}{\left(\rho_{m}-\rho_{s}\right)}^{2}P\left(q\right)S_{P-Y}\left(q\right)$$

Being *P*(*q*) and *S_P-Y_*(*q*) the form and structure factor (hard sphere with Percus–Yevick closure) of polydisperse interacting spheres (*R*~10 nm), respectively [23,24,25].

### 3.4. Quartz Crystal Microbalance With Dissipation (QCM-D)

QCM-D measurements were performed at PSCM laboratories (EPN campus, Grenoble) using a commercial apparatus (E4 Q-Sense, Biolin Scientific AB, Vaästra Froölunda, Sweden) which allows simultaneous measurements of the changes in the resonance frequency (Δ*f*) and in the energy dissipation (Δ*D*) due to molecular adsorption process. Sensors with a silica surface (QSX 303 silicon dioxide, 50 nm) were employed, in order to have an analogous surface of NR experiments. Sensors were cleaned with water and ethanol according to standard procedures reported elsewhere [34]. 

### 3.5. Neutron Reflectivity

Neutron reflectivity data were acquired at the MARIA neutron reflectometer [50] operated by Jülich Centre for Neutron Science at Heinz Maier-Leibnitz Zentrum in Garching (Germany), using custom temperature-regulated liquid cells [51]. The measurements were performed using two different wavelengths, 10 Å for the low-q region and 5 Å for the high-q region up to 0.25 Å^−1^, with a 10% wavelength spread. The change of solvent contrast in the liquid cells was performed using a combination of valves and a peristaltic pump, at small flow rates ~0.5 mL/min. In a reflectivity experiment, a beam was sent to the sample at grazing angle and the reflected intensity R was collected as a function of the reflection angle momentum transfer perpendicular to the interface *q_z_* (*q_z_* = 4πsinθ/λ, where θ and λ are the angle of the incident beam and wavelength, respectively). 

Reflectivity is related to the scattering length density (SLD) across the interface by the relation:

*R*(*q_z_*) ~ (16π^2^)|*ρ*(*q_z_*)|^2^/*q_z_*^2^ (11)

valid in the Born approximation [52]. *ρ*(*q_z_*), that is the Fourier transform of the scattering length density profile *ρ*(*z*) along the normal to the interface, gives information about structure and composition of each layer. The scattering length density is given by:

*ρ*(*z*) = ∑*j bjnj* (12)

where *n_j_* is the number of nuclei per unit volume and *b_j_* is the scattering length of nucleus *j*. The method of analysis used for specular reflection data involves modeling the interface as a series of parallel layers, each of them characterized by an average scattering length density and a thickness. These parameters are used to calculate a model reflectivity profile by means of the optical matrix method [53]. The interfacial roughness between two consecutive layers is included in the model by the Abeles method, as described by Nevot and Croce [53]. The calculated profile is compared to the measured profile and the quality of the fit is assessed by using χ^2^ in the least-squares method. 

The technique allows the collection of structural information about the different layers of the membrane [54,55,56,57]. In particular, if the silicon support and the bulk water have been seen as bulk infinite layers, the silicon oxide layer, the water layer between the silicon oxide, and the membrane and the different hydrophilic and hydrophobic layers of the lipid membranes have been modelled as defined layers with a proper thickness, compactness, and mean composition (and therefore contrast). Reflectivity has been measured from the silicon supports and the samples in different water solutions (H_2_O and D_2_O) with different contrasts for neutrons analysed by a contemporary fit, performed by the MotoFit program [58].

## 4. Conclusions

Our work was focused on the development of a model mucosal environment to deepen the understanding of the interactions of mucus with biomolecules on the nanometer scale. We mimicked mucus by its major component, mucin, and we investigated its interaction with a bio-inspired polymer, d,l-ARGO7. NR experiments on mucin thin film and on mucin layer adhered on top of complex phospholipid bilayer are shown to be optimal techniques to study the stability of complexes and the mucus penetration attitude of biomacromolecules. 

The study identifies d,l-ARGO7, having pH tunable physical-chemical properties, as a good candidate to interact with mucin in physiological conditions, by forming some tight clusters of hundreds of nanometer size in the bulk. Beside these phase-separated aggregates, SAXS investigation suggested that some loose entanglement occurs between mucins and the polymers, affecting the mesh structure in bulk. Addressing mucin thin film study, complementary investigation by QCM-D and NR showed that thin mucin layers (20 Å thick) can be deposited on the silicon substrate and that they are very stable under experimental treatments. In addition, they are suitable for structural investigation also in interaction with macromolecules that may affect their properties. In particular d,l-ARGO7 was found to interact first with the more external mucin moiety, exposed to the bulk solvent where the polymers come from, and to slightly deteriorate the layer, probably due to the formation of the complexes such as those elucidated by SAXS in the bulk.

A very important achievement was the successful building up of a model for mucus-coated epithelial tissues by the deposition of a mucin layer on the top of a glycolipid containing phospholipid single membrane, deposited on a silicon substrate. This model is quite complex, not only because of the achieving of the mucin deposition on top but also because of the complex composition and asymmetric disposition of the underlying membrane. The bio membrane model is stable under experimental treatments, such as solvent exchange and polymer interaction, and has been found to be suitable for investigations with surface techniques, such as neutron reflectometry. We could, in fact, study the cross structural details of the mucus-covered epithelium model in interaction with d,l-ARGO7 within the Å resolution. d,l-ARGO7 was proved to make complexes with mucin on the top of the lipid bilayer, affecting the stability of the deposited material, eventually causing its partial removal by a final rinsing procedure. This behaviour was consistent with what was found in the simpler system of a single mucin layer deposited on silicon. Indeed, surface experiments allow performing a rinsing process of the bulk solution after the interaction among the incoming molecules and mucin chains has occurred, which is not achievable in bulk model investigation. This is an indirect way to unveil the physical outcome of the molecules/mucin bound, determining either a phase separation and disentanglement of the mucin mesh or a re-enforcing of the net itself. Results let us identifying d,l-ARGO7 as a possible mucin sequestering agent capable of inducing defects to the deposited hydrogel becoming prone to water penetration. This effect is opposite to the mesh strengthening play claimed for other muco-adhesive molecules, such as chitosan [22,32]. In this perspective, the role of ARGO7 PAAs may be of relevance, considering the serious implications of a defective muco-clearance in many pathologies, such as cystic fibrosis, where patients suffer for the disfunction of the muco-clearance, causing aberrant production of mucus in the lungs.

The study is involved in topical drug delivery vector design and opens the way to the detailed investigation of the fate of different macromolecules during their crossing of the mucosal barrier, facing the epithelial cells surface, and eventually interacting with the cell membrane.

## Supplementary Materials

Supplementary materials can be found at https://www.mdpi.com/1422-0067/20/15/3712/s1.
