# Supplementary material for: Mucin Thin Layers: A Model for Mucus-Covered Tissues
Source: Int J Mol Sci. 2019 Jul 29;20(15):3712. doi: 10.3390/ijms20153712 (PMC6695901; doi:10.3390/ijms20153712)
Supplement: Supplementary file 1 [file ijms-20-03712-s001.pdf]

Supplementary Material for the manuscript

Mucin thin layers: a model for mucus covered tissues

Valeria Rondelli [1,*], Emanuela Di Cola [1], Alexandros Koutsioubas[2], Jenny Alongi[3], Paolo Ferruti[3], Elisabetta Ranucci [3] and Paola Brocca[1]

[1] Università degli Studi di Milano, department of Medical Biotechnologies and Traslational Medicine; valeria.rondelli@unimi.it
[2] Jülich Centre for Neutron Science (JCNS) at Heinz Maier-Leibnitz Zentrum (MLZ), Forschungszentrum Jülich GmbH, Lichtenbergstrasse 1, 85748 Garching, Germany; a.koutsioumpas@fz-juelich.de
[3] Università degli Studi di Milano,department of Chemistry; elisabetta.ranucci@unimi.it

* Correspondence: valeria.rondelli@unimi.it; Tel.: +39-0250330323

Table S1. Fit parameters of the mucin layer deposited on the silicon surface. Parameters correspond to a contemporary fit performed on H2O and D2O solutions with 150mM NaCl. Errors have been estimated by changing the parameters up to a variation of two in the χ2.

	Thickness (±1 Å)	SLD (± 0.05*10^{-6}Å^{-2})	Solv p (±5%vol)	Roughness (±2Å)
Mucin	21	2.5 (H_2O)<br>5.6 (D_2O)	40	3

Table S2. Fit parameters of the mucin layer deposited on the silicon surface, after the interaction with ARGO7-LD. Parameters correspond to a contemporary fit performed on H2O and D2O solutions with 150mM NaCl. Errors have been estimated by changing the parameters up to a variation of two in the χ2.

	Thickness (±1 Å)	SLD (± 0.05*10^{-6}Å^{-2})	Solv p (±5%vol)	Roughness (±2Å)
Mucin	21	2.5 (H_2O)<br>5.6 (D_2O)	45	2
ARGO7-LD	70	2	90	5

Table S3. Fit parameters of the mucin layer deposited on the silicon surface, after flushing ARGO7-LD. Parameters correspond to a contemporary fit performed on H2O and D2O solutions with 150mM NaCl. Errors have been estimated by changing the parameters up to a variation of two in the χ2.

	Thickness (±1 Å)	SLD (± 0.05*10^{-6}Å^{-2})	Solv p (±5%vol)	Roughness (±2Å)
Mucin	20	2.5 (H_2O)<br>5.6 (D_2O)	50	2

A)

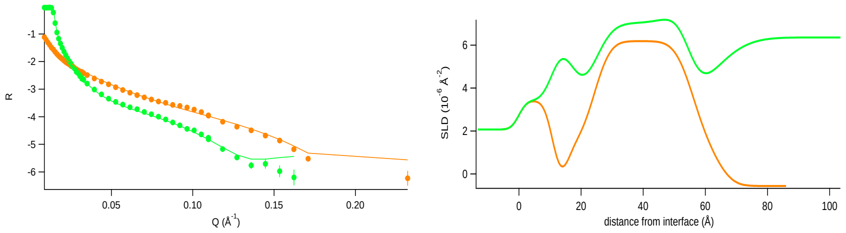

B)

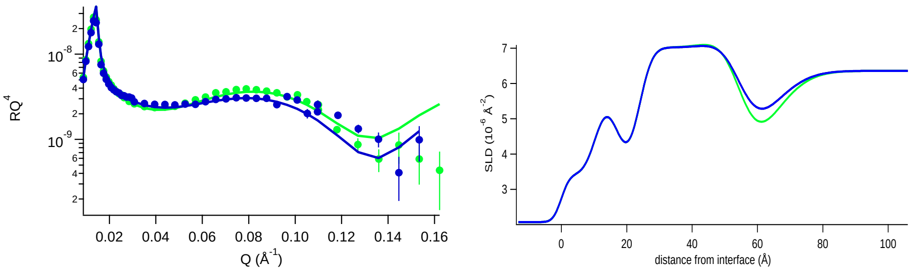

C)

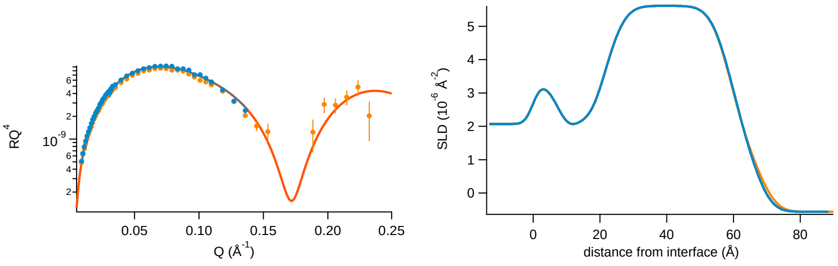

Figure S1. Reflectivity curves (left panel, symbols),relative best fits (left panel, lines) and obtained SLD profiles (right panel) of A) the DPPC membrane investigated in H_2O NaCl (orange), in D_2O NaCl (green); B) the DPPC membrane D_2O NaCl investigated before (green) and after (blue) the interaction with mucin; C) the DPPC membrane H_2O NaCl investigated before (orange) and after (sky blue) the interaction with mucin.

Table S4. Fit parameters of the d_{62}DPPC membrane at room temperature. Parameters correspond to a contemporary fit performed on H2O and D2O solutions with 150mM NaCl. Errors have been estimated by changing the parameters up to a variation of two in the $\chi 2$. For each parameter, the maximum error found was kept.

	**Thickness (±1 Å)**	**SLD (± 0.05*10^{-6}Å^{-2})**	**Solv p (±5%vol)**	**Roughness (±2Å)**
solvent	1			3
Heads in	9	1.75	32	4
Chains in	18	7.66	25	4
Chains out	20	7.66	25	4
Heads out	10	1.75	32	5

Table S5. Fit parameters of the d_{62}DPPC membrane after the interaction with mucin. Parameters correspond to a contemporary fit performed on H2O and D2O solutions with 150mM NaCl. Errors have been estimated by changing the parameters up to a variation of two in the χ2. For each parameter, the maximum error found was kept.

	**Thickness (±1 Å)**	**SLD (± 0.05*10^{-6}Å^{-2})**	**Solv p (±5%vol)**	**Roughness (±2Å)**
solvent	1			3
Heads in	9	1.75	32	4
Chains in	18	7.66	25	4
Chains out	20	7.66	25	4
Heads out	8	1.95 (H_2O)<br>3 (D_2O)	32	4

Table S6. Fit parameters of the d_{62}DPPC-GM1 membrane at room temperature. Parameters correspond to a contemporary fit performed on H2O and D2O solutions with 150mM NaCl. Errors have been estimated by changing the parameters up to a variation of two in the χ2. For each parameter, the maximum error found was kept.

	**Thickness (±1 Å)**	**SLD (± 0.05*10^{-6}Å^{-2})**	**Solv p (±5%vol)**	**Roughness (±2Å)**
solvent	5			3
Heads in	7	1.75	20	5
Chains in	16	7.11	8	5
Chains out	17	6.54	8	5
Heads out	6	1.78	20	5

Table S7. Fit parameters of the d_{62}DPPC-GM1 membrane after the deposition of mucin on top. Parameters correspond to a contemporary fit performed on H2O and D2O solutions with 150mM NaCl. Errors have been estimated by changing the parameters up to a variation of two in the χ2. For each parameter, the maximum error found was kept.

	**Thickness (±1 Å)**	**SLD (± 0.05*10^{-6}Å^{-2})**	**Solv p (±5%vol)**	**Roughness (±2Å)**
solvent	0			3
Heads in	9	1.75	55	4
Chains in	18	7.11	45	5
Chains out	18	6.54	45	5
Heads out	10	1.78	55	5
Mucin	22	2.5 (H_2O)<br>5.6 (D_2O)	63	7

Table S8. Fit parameters of the d_{62}DPPC-GM1 + mucin model system after interaction with ARGO7-LD. Parameters correspond to a contemporary fit performed on H2O and D2O solutions with 150mM NaCl. Errors have been estimated by changing the parameters up to a variation of two in the $\chi 2$. For each parameter, the maximum bar found was kept.

	**Thickness (±1 Å)**	**SLD (± 0.05*10^{-6}Å^{-2})**	**Solv p (±5%vol)**	**Roughness (±2Å)**
solvent	0			3
Heads in	9	1.75	55	4
Chains in	18	7.11	45	5
Chains out	18	6.54	45	5
Heads out	10	1.78	55	5
Mucin	40	2.5 (H_2O)<br>5.6 (D_2O)	70	8
ARGO7-LD	50	2	90	8

Table S9. Fit parameters of the d_{62}DPPC-GM1 + mucin model system after flushing ARGO7-LD. Parameters correspond to a contemporary fit performed on H2O and D2O solutions with 150mM NaCl Errors have been estimated by changing the parameters up to a variation of two in the $\chi 2$. For each parameter, the maximum bar found was kept.

	**Thickness (±1 Å)**	**SLD (± 0.05*10^{-6}Å^{-2})**	**Solv p (±5%vol)**	**Roughness (±2Å)**
solvent	0			3
Heads in	9	1.75	55	4
Chains in	18	7.11	45	5
Chains out	18	6.54	45	5
Heads out	10	1.78	55	5
Mucin	25	2.5 (H_2O)<br>5.6 (D_2O)	80	8
